# Additive Uncorrelated Relaxed Clock Models for the Dating of Genomic Epidemiology Phylogenies

**DOI:** 10.1093/molbev/msaa193

**Published:** 2020-07-28

**Authors:** Xavier Didelot, Igor Siveroni, Erik M Volz

**Affiliations:** 1 School of Life Sciences, University of Warwick, Coventry, United Kingdom; 2 Department of Statistics, University of Warwick, Coventry, United Kingdom; 3 Department of Infectious Disease Epidemiology, School of Public Health, Imperial College London, London, United Kingdom

**Keywords:** clock model, dated phylogeny, genomic epidemiology

## Abstract

Phylogenetic dating is one of the most powerful and commonly used methods of drawing epidemiological interpretations from pathogen genomic data. Building such trees requires considering a molecular clock model which represents the rate at which substitutions accumulate on genomes. When the molecular clock rate is constant throughout the tree then the clock is said to be strict, but this is often not an acceptable assumption. Alternatively, relaxed clock models consider variations in the clock rate, often based on a distribution of rates for each branch. However, we show here that the distributions of rates across branches in commonly used relaxed clock models are incompatible with the biological expectation that the sum of the numbers of substitutions on two neighboring branches should be distributed as the substitution number on a single branch of equivalent length. We call this expectation the additivity property. We further show how assumptions of commonly used relaxed clock models can lead to estimates of evolutionary rates and dates with low precision and biased confidence intervals. We therefore propose a new additive relaxed clock model where the additivity property is satisfied. We illustrate the use of our new additive relaxed clock model on a range of simulated and real data sets, and we show that using this new model leads to more accurate estimates of mean evolutionary rates and ancestral dates.

## Introduction

Epidemiological analysis of pathogen genomic data often relies on the construction and interpretation of dated phylogenies. Dated phylogenies have branch lengths measured in units of time (e.g., years or days) instead of genetic distance. The leaves of a dated phylogeny are aligned on the time axis with their isolation dates (which are usually known), and the internal nodes are aligned with the time when the corresponding common ancestors existed which is usually unknown but can be estimated. Time-scaled phylogenetic analysis represents a very useful and popular tool for genomic epidemiology, allowing researchers to study population size dynamics (Ho and Shapiro 2011), transmission (Didelot et al. 2017), pathogen population structure (Volz et al. 2020), or host population structure (Volz et al. 2013). Dated phylogenies can be built directly from the genetic data using Bayesian phylogenetic methods implemented in BEAST (Suchard et al. 2018; Bouckaert et al. 2019). Alternatively, a two-step approach can be used, which is based on firstly building a standard phylogeny and secondly estimating the date of each node in this phylogeny. The first step (standard phylogenetics) can be performed, for example, using RAxML (Stamatakis 2014), PhyML (Guindon et al. 2010), FastTree (Price et al. 2010), or IQ-TREE (Nguyen et al. 2015). The second step (phylogeny dating) can be performed, for example, using LSD (To et al. 2016), node.dating (Jones and Poon 2017), treedater (Volz and Frost 2017), TreeTime (Sagulenko et al. 2018), or BactDating (Didelot et al. 2018). In this article, for ease of presentation, we initially focus on the two-step phylogeny dating approach, and later show how our findings are applicable to the integrated approach too.

An important consideration when building a dated phylogeny is the choice of the clock model, which represents the way in which mutations accumulate during the evolution of the population (Kumar 2005; Lepage et al. 2007; Drummond and Suchard 2010; Lartillot et al. 2016). In the phylogeny dating approach, the clock model represents the stochastic relationship, for each branch *i* of the phylogeny, the duration *l_i_* separating the nodes at the top and bottom of the branch, and the number *x_i_* of mutations that occurred on the branch. The simplest clock model is called the strict clock (SC) model and assumes a constant rate *μ* of mutation on all the branches (Zuckerkandl and Pauling 1962). Therefore, a branch of duration *l_i_* will contain a number of mutations *x_i_* which is Poisson distributed with parameter $\mu l_{i}$. The SC model (Zuckerkandl and Pauling 1962) has just a single parameter *μ* and this simplicity is attractive, but it is often too simple because of variations in the mutation rate from one lineage to another.

A number of alternatives to the SC model have been proposed, with by far the most popular being the uncorrelated relaxed clock (RC) model (Drummond et al. 2006). Under this model, each branch has its own mutation rate *m_i_*, and these per-branch rates are independent of one another. In current implementations of the uncorrelated RC model, the rates *m_i_* are drawn independently and identically from a well-defined rate distribution, for example, a lognormal distribution (Drummond et al. 2006), an exponential distribution (Drummond et al. 2006; To et al. 2016), a normal distribution (Sagulenko et al. 2018), or a gamma distribution (Volz and Frost 2017; Didelot et al. 2018). However, we found that the use of the same distribution for all per-branch rates of the uncorrelated RC model is inconsistent with the intuitive biological expectation of additivity between branches of the phylogeny. For example, if we consider two branches *i* and *j* of the tree with length *l_i_* and *l_j_*, respectively, then the distribution of $x_{i}+x_{j}$ is not the same as the distribution for a branch of length $l_{i}+l_{j}$. The currently used models are therefore not robust to adding or removing genomes in the phylogeny, since the way these genomes find common ancestors with the remaining genomes will cause some branches to be split or merged. The nonadditivity property of frequently used RC models becomes clear when we consider splitting or merging branches of the tree. But, it is also important even if there is no intention to add or remove genomes, since it means that the dating results are not robust to the selection of genomes used for analysis.

Using an additive model is likely to be especially important for applications of dating in genomic epidemiology where many branches of short duration are considered, due to very large sample sizes and epidemic processes of interest sometimes occurring in a matter of days (Carroll et al. 2015; Faria et al. 2017). It is also very relevant to real-time studies of pathogen outbreaks, where new cases are continuously added onto the phylogeny over time, splitting ancestral branches (Quick et al. 2016; Dinh et al. 2018; Fourment et al. 2018; Hadfield et al. 2018; Gill et al. 2020). Here, we propose alternative robust uncorrelated RC models which solve this issue and therefore have better statistical and biological properties compared with the current models. We consider both the case where the number of mutations on a branch is discrete or continuous. We illustrate the difference between our models and previous models using simulations, and show that previous models can lead to misleading conclusions on both simulated and real genomic epidemiology data sets.

## New Approaches

### Additivity of the SC Model

We start with the simple SC model (Zuckerkandl and Pauling 1962) in order to set notations and define the additivity property in this context. Under the SC model, we have that each branch mutates as a Poisson process with rate *μ*. The discrete number of mutations *x_i_* on a branch of duration *l_i_* (which could be measured in years or days, etc.) is therefore:

(1) $$x_{i}∼\text{Poisson}(l_{i}\mu ).$$

Note that we use lower case symbols for both random variables and their realizations, which is a frequently used abuse of notation in the field (and also more generally when Greek symbols are used). Let us now consider two branches of lengths *l*_1_ and *l*_2_. Under the SC model, the distribution of the convolution $x_{1}+x_{2}$, that is, the sum of the number of mutations on both branches, is the same as the distribution of the number *x* of mutations on a branch of length $l=l_{1}+l_{2}$, because:

(2) $$x_{1}\sim \text{Poisson}(l_{1}\mu )\;\text{and}\;x_{2}\sim \text{Poisson}(l_{2}\mu )\;\Rightarrow x_{1}+x_{2}\sim \text{Poisson}((l_{1}+l_{2})\mu ).$$

We call this property the additivity of the SC model, and note that it is a consequence of the infinite divisibility of the Poisson distribution.

### Nonadditivity of Previous Uncorrelated RC Models

The uncorrelated RC model was first proposed by Drummond et al. (2006). In this model, each branch has its own mutation rate *m_i_*. A convenient choice for the distribution of the *m_i_* rates is a Gamma(k,θ) distribution, since this is the conjugate of the Poisson distribution of *x_i_* given *l_i_* (note that here and throughout this article, we use a shape-scale parametrization of the gamma distribution). As previously noted (Volz and Frost 2017), this choice leads to:

(3) $$x_{i}∼\text{NegBin}\left(k,\frac{\theta l_{i}}{1+\theta l_{i}}\right).$$

More generally, let *μ* and $\sigma^{2}$ denote the mean and variance of the distribution of per-branch rates *m_i_*. In the case of the Gamma(k,θ) distribution, this is achieved by setting $k=\frac{\mu^{2}}{\sigma^{2}}$ and $\theta =\frac{\sigma^{2}}{\mu }$. Using the laws of total expectation and variance of *x_i_*, we can show that:

(4) $$E(x_{i})=E(E(x_{i}|m_{i}l_{i}))=E(m_{i}l_{i})=\mu l_{i},$$

(5) $$V(x_{i})=E(V(x_{i}|m_{i}l_{i}))+V(E(x_{i}|m_{i}l_{i}))=E(m_{i}l_{i})+V(m_{i}l_{i})=\mu l_{i}+\sigma^{2}l_{i}^{2}.$$

We note that the expectation is the same as in the SC model, whereas the variance is increased by an additive factor $\sigma^{2}l_{i}^{2}$. The fact that the variance is increased makes sense since RC is a relaxation of the SC model. However, the variance is increased by a factor that is not proportional to the branch length *l_i_*, and this implies that the model does not have the additivity property. In particular, we find that the variance of the number of mutations *x* on a branch of length $l=l_{1}+l_{2}$ is greater than the variance of $x_{1}+x_{2}$ where *x*_1_ and *x*_2_ are numbers of mutations on branches of lengths *l*_1_ and *l*_2_, respectively:

(6) $$V(x)=\mu l+\sigma^{2}l^{2}\;>V(x_{1}+x_{2})=\mu (l_{1}+l_{2})+\sigma^{2}(l_{1}^{2}+l_{2}^{2}).$$

Since the variances of *x* and $x_{1}+x_{2}$ are not the same, their distributions are clearly not identical and so the RC is not additive like the SC model. This is true for the RC model in equation (3) which is based on the same gamma distribution for all per-branch rates, but the calculation above was not based on any particular distribution, so that it also applies to any other RC model based on any other identical distribution for the per-branch rates. The fact that the RC model does not have the additivity property is problematic both from a statistical and biological point of view.

### Additive Uncorrelated RC Model

In order to obtain the additivity property in a RC model, we propose an alternative model which we call the additive RC (ARC) model. This model has parameters *μ* and *ω* such that a branch of duration *l_i_* has mutation rate *m_i_* with expectation $E(m_{i})=\mu$ and variance $V(m_{i})=\mu \omega /l_{i}$. Using the laws of total expectation and variance as previously, we find that:

(7) $$E(x_{i})=E(E(x_{i}|m_{i}l_{i}))=E(m_{i}l_{i})=\mu l_{i},$$

(8) $$V(x_{i})=E(V(x_{i}|m_{i}l_{i}))+V(E(x_{i}|m_{i}l_{i}))=E(m_{i}l_{i})+V(m_{i}l_{i})=\mu l_{i}(1+\omega ).$$

The expected number of mutations under the ARC model is therefore the same as in the SC model and RC model. The variance is increased relative to the SC model by a multiplicative factor 1+ω. The values of the expectation and variance on the number of mutations are therefore compatible with the desired additivity property of the proposed model. However, this is a necessary but not sufficient condition. For the model to be additive, we need the distributions to be additive, not just their expectations and variances. We can obtain this full additivity property using a gamma distribution for the mutation rate *m_i_* of a branch of length *l_i_* as follows:

(9) $$m_{i}∼\text{Gamma}\left(\frac{\mu l_{i}}{\omega },\frac{\omega }{l_{i}}\right).$$

Since the gamma distribution is the conjugate prior to the Poisson $\left(m_{i}l_{i}\right)$ distribution of *x_i_* given *m_i_*, we get:

(10) $$x_{i}∼\text{NegBin}\left(\frac{\mu l_{i}}{\omega },\frac{\omega }{1+\omega }\right).$$

This ARC model clearly satisfies the additivity property, since the sum of two negative binomial random variables with the same second parameter is also a negative binomial random variable. Specifically:

(11) $$\begin{array}{rcl} & & x_{1}\sim \text{NegBin}\left(\frac{\mu l_{1}}{\omega },\frac{\omega }{1+\omega }\right)\\ & & \;\text{and}\;x_{2}\sim \text{NegBin}\left(\frac{\mu l_{2}}{\omega },\frac{\omega }{1+\omega }\right)\\ & \Rightarrow & x_{1}+x_{2}\sim \text{NegBin}\left(\frac{\mu (l_{1}+l_{2})}{\omega },\frac{\omega }{1+\omega }\right). \end{array}$$

Like the Poisson distribution used in the SC model (eq. 1), the negative binomial distribution used here (i.e., with a constant second parameter $\frac{\omega }{1+\omega }$) is infinitely divisible for its first parameter which is proportional to the branch length.

### Continuous RC Models

In this section, we consider models in which the branch lengths *x_i_* of the phylogenetic tree, measured in units of substitutions, are continuous. This is useful because most standard phylogenetic software return trees where branch lengths are continuous, in order to accommodate uncertainties in ancestral sequence reconstructions (Yang and Rannala 2012) and to account for nonuniform mutation models which give different weights to different types of mutations (Liò and Goldman 1998). Gamma distributions are a natural choice for this as previously noted (Didelot et al. 2018). For example, in the case of a continuous SC (cSC) model with rate *μ*, instead of the discrete Poisson distribution from equation (1), we can use the gamma distribution with the same expectation and variance, namely:

(12) $$x_{i}∼\text{Gamma}(\mu l_{i},1).$$

This cSC model satisfies the additivity property, since:

(13) $$x_{1}\sim \text{Gamma}(\mu l_{1},1)\;\text{and}\;x_{2}\sim \text{Gamma}(\mu l_{2},1)\;\Rightarrow x_{1}+x_{2}\sim \text{Gamma}(\mu (l_{1}+l_{2}),1).$$

(13)

A continuous uncorrelated RC (cRC) model was recently proposed (Didelot et al. 2018) based on the assumption that each branch has its own mutation rate *m_i_* with mean *μ* and variance $\sigma^{2}$, as in the discrete RC model. Specifically, *x_i_* was proposed to be gamma distributed as follows:

(14) $$x_{i}∼\text{Gamma}\left(\frac{\mu^{2}l_{i}}{\mu +\sigma^{2}l_{i}},1+\frac{\sigma^{2}l_{i}}{\mu }\right).$$

This choice is analogous to the discrete RC model (Drummond et al. 2006) previously mentioned, and suffers from the same issue of nonadditivity. In particular, we can use the laws of total expectation and variance of *x_i_* to get $E(x_{i})=\mu l_{i}$ and $V(x_{i})=\mu l_{i}+\sigma^{2}l_{i}^{2}$ exactly as in the discrete case (cf. eqs. 4 and 5). If $\sigma^{2}=0$ this model reduces to the cSC model (eq. 12) which is additive, but otherwise this model does not have the additivity property. This is true for the cRC model in equation (14) but also for any other cRC model that assumes that the per-branch rates are independent and identically distributed.

We can remedy this issue in a similar way as we did for the discrete case, and define a continuous additive RC (cARC) model. We consider the model with parameters *μ* and *ω* such that a branch of duration *l_i_* has mutation rate *m_i_* with the same expectation and variance as in the discrete case, that is, $E(m_{i})=\mu$ and variance $V(m_{i})=\mu \omega /l_{i}$. By application of the laws of total expectation and variance, we get the same expectation and variance for *x_i_* as in the discrete case, compare equations (7) and (8). These formulas for the expectation and variance of *x_i_* are necessary for the additivity of the model, but as noted in the discrete case they are not sufficient since we also need the distributions themselves to be additive. To obtain this property, we define the cARC using the following gamma distribution:

(15) $$x_{i}∼\text{Gamma}\left(\frac{\mu l_{i}}{1+\omega },1+\omega \right).$$

If *ω *= 0, this model reduces to the cSC model (eq. 12). The cARC model has the additivity property since the sum of two gamma-distributed random variables with the same scale parameter is also gamma distributed with the same scale. Specifically:

(16) $$\begin{array}{rcl} & & x_{1}\sim \text{Gamma}\left(\frac{\mu l_{1}}{1+\omega },1+\omega \right)\\ & & \;\text{and}\;x_{2}\sim \text{Gamma}\left(\frac{\mu l_{2}}{1+\omega },1+\omega \right)\\ & \Rightarrow & x_{1}+x_{2}\sim \text{Gamma}\left(\frac{\mu (l_{1}+l_{2})}{1+\omega },1+\omega \right). \end{array}$$

Note that there is a difference in the way we derived this continuous model (cARC, eq. 15) compared with the discrete model (ARC, eq. 10): In the latter we selected a distribution on *m_i_* to deduce the distribution of *x_i_*, whereas in the former we selected a distribution of *x_i_* directly, without worrying about the distribution of *m_i_* (which are not identically distributed). There is however no difference in practice between these two approaches: In the discrete case, the distribution of *m_i_* was selected to get the distribution of *x_i_* we wanted (i.e., with the additivity property) which is not statistically more principled than directly specifying the distribution of *x_i_*.

## Results

### Comparison of Model Properties

**Fig. 1.**
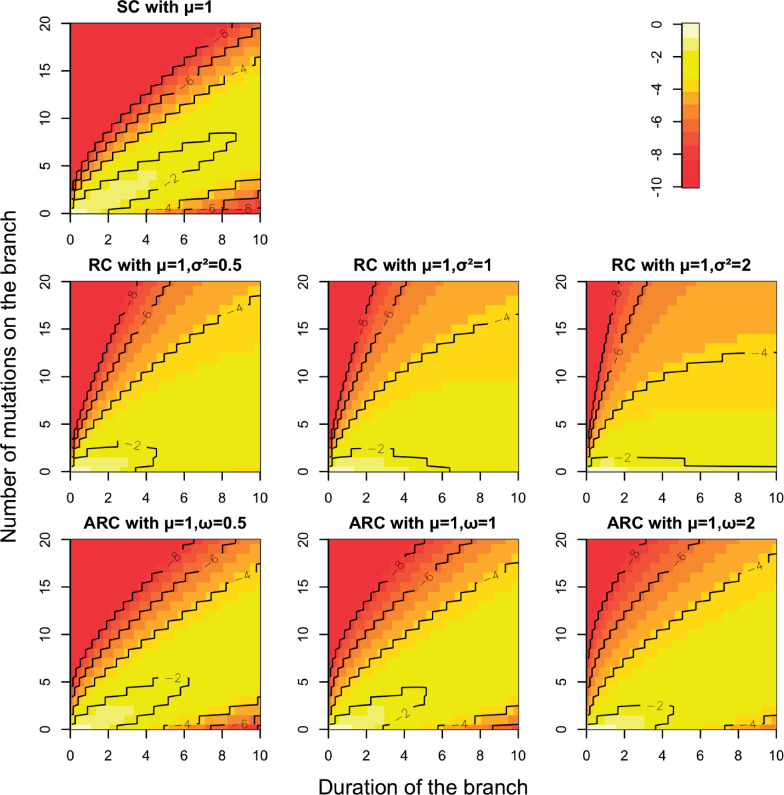
Comparison of clock models for discrete branch lengths. The top-left plot shows the SC model, with *μ* = 1. The second row shows the RC model, with *μ* = 1 and $\sigma^{2}=0.5$, 1, and 2, respectively, from left to right. The third row shows the ARC model, with *μ* = 1 and ω=0.5, 1, and 2, respectively, from left to right. In each plot, the *x*-axis shows values of *l_i_*, the *y*-axis shows values of *x_i_* and the color represents the value of the log of $p(x_{i}|l_{i})$ as per the legend.

The six clock models described above and their properties are summarized in table 1. We compared the discrete distributions of the number of substitutions implied by the SC model, the RC model, and the new ARC model, varying both the duration of the branches considered and the level of relaxation in the RC and ARC models. Specifically, the distributions of the number of substitutions *x_i_* on a branch of duration *l_i_* are shown in figure 1 for the SC model (eq. 1), the RC model (eq. 3), and the ARC model (eq. 10). Increasing the variance of the per-branch rates in the RC model (parameter $\sigma^{2}$) and the ARC model (parameter *ω*) made the distributions of substitutions increasingly diffuse relative to the SC model, as expected. There are however marked differences in behavior between the RC and ARC models: In the RC model, the distribution mode for longer branches quickly shifts to small values as relaxation is increased, whereas this is not the case in the ARC model. Conversely, for short branches, even a high $\sigma^{2}$ in the RC model does not imply much relaxation, whereas a high *ω* in the ARC model has a much clearer effect for small branches. On a branch of length *l_i_*, the excess variance in the number of mutations of the RC model relative to the SC model is $\sigma^{2}l_{i}^{2}$ (eq. 5), whereas in the ARC model it is $\mu l_{i}\omega$ (eq. 8). If we set *μ *= 1 and $\sigma^{2}=\omega$, we, therefore, have that the variance is greater in the ARC model than in the RC model for branches of length $l_{i}<1$ and vice versa for branches of length $l_{i}>1$, as can be seen by comparison of the last two rows of figure 1.

**Table 1.** Summary of the Six Clock Models under Study and Their Properties.

Model	Full Name	Relaxed	Additive	Continuous	Equation	References
SC	Strict clock	N	Y	N	1	Zuckerkandl and Pauling (1962)
RC	Relaxed clock	Y	N	N	3	Drummond et al. (2006)
ARC	Additive relaxed clock	Y	Y	N	10	This study
cSC	Continuous strict clock	N	Y	Y	12	Didelot et al. (2018)
cRC	Continuous relaxed clock	Y	N	Y	14	Didelot et al. (2018)
cARC	Continuous additive relaxed clock	Y	Y	Y	15	This study

We performed a similar comparison for the models using continuous distributions of the number of substitutions on each branch. The distributions of number of mutations *x_i_* on a branch of duration *l_i_* are shown in supplementary figure S1, Supplementary Material online for the strict cSC model (eq. 12), the relaxed cRC model (eq. 14), and the new cARC model (eq. 15). We note that these results are very similar to the discrete case for all six models considered, that is, SC versus cSC, RC versus cRC, and ARC versus cARC (compare fig. 1 and supplementary fig. S1, Supplementary Material online). This indicates that the gamma distributions used in the three continuous models are good continuous equivalents to the Poisson and negative binomial distributions used in the three discrete models. In particular, comparison between cRC and ARC shows very similar features to the ones described above between RC and ARC concerning the effect on short versus long branches. In the discrete situation, the SC model defined in equation (1) is not a special case of the RC model defined in equation (3). However, in the continuous situation, we have a useful property that the cSC model defined in equation (12) is a special case of both the cRC model (by setting $\sigma^{2}=0$ in eq. 14) and the cARC model (by setting *ω *= 0 in eq. 15). This property is useful for model selection, since it means that the cSC model is embedded within the cRC and the cARC models.

### Application to Simulated Data Sets

We simulated 100 data sets, each of which consisted of 100 genomes of 10,000 bp sampled at regular intervals between 2010 and 2020. The ARC model was used to simulate mutations along the branches of this dated phylogeny, with a mean rate of *μ* = 5 mutations per genome per year, and a relaxation parameter varying between *ω* = 0 (in which case the model reduced to the SC model) and *ω* = 10. Undated phylogenies were reconstructed from the genomes using PhyML (Guindon et al. 2010) which were used as input trees in BactDating (Didelot et al. 2018). Separate MCMC runs were performed assuming either the old RC model or the new ARC model. Each MCMC was run for 10^5^ iterations which took ∼10 min on a single core of a standard desktop computer. Good convergence and mixing properties of the MCMC results were found using both the Gelman–Rubin diagnostic (Gelman and Rubin 1992; Brooks and Gelman 1998) and an effective sample size test implemented in CODA (Plummer et al. 2006).

**Fig. 2.**
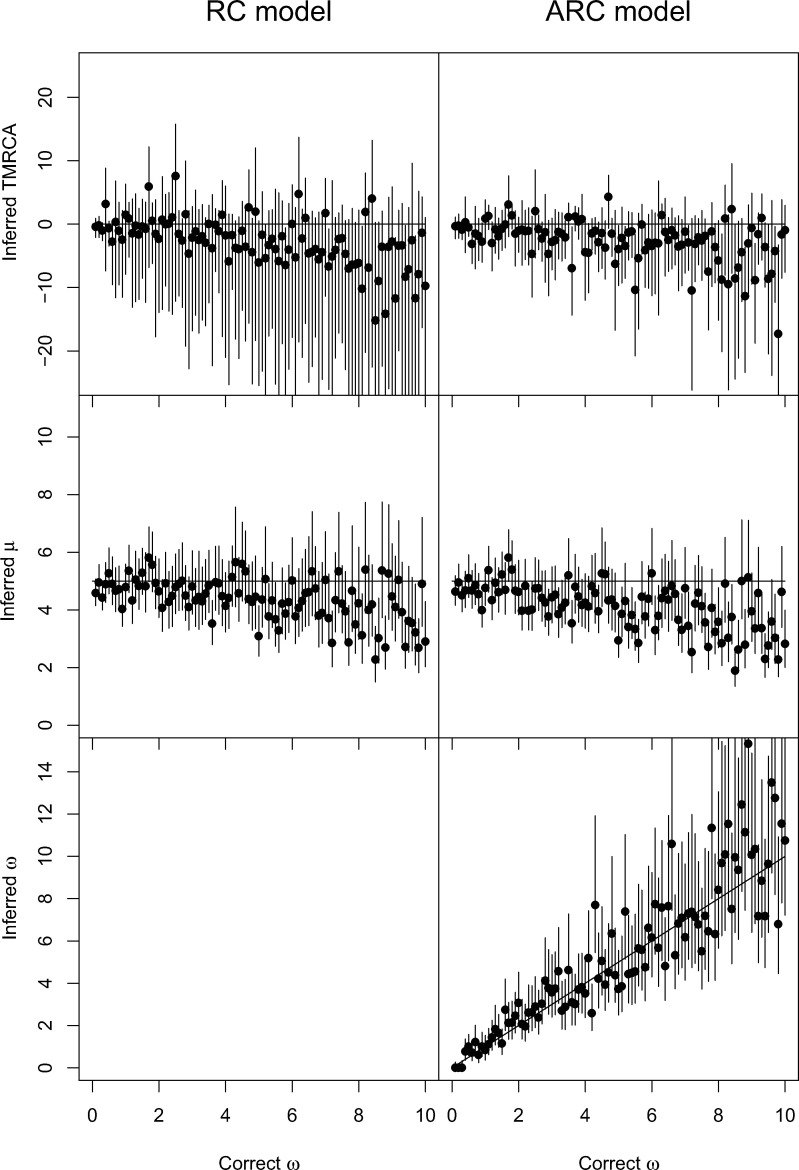
Application of BactDating to 100 simulated data sets. On the left, inference used the RC model and on the right, the ARC model. The top row shows inferred values of the TMRCA (relative to the correct value), the middle row shows inferred values of the mean mutation rate *μ*, and the bottom row shows inferred values of the relaxation parameter *ω* for the ARC model. In each plot, the *x*-axis represents the value of *ω* used in the simulations (varied between 0 and 10) and the *y*-axis represents the inferred values, with a dot for the posterior mean and a bar for the 95% credible interval.

We compared the fit of these two models by computing the deviance information criterion (DIC) of both models (Spiegelhalter et al. 2002). We found that the ARC had significantly better fit (i.e., smaller DIC) for all simulations with ω>1, which is as expected because the data were simulated from the ARC model. This model comparison was more ambiguous when ω<1, which again is as expected since when *ω* is close to zero both the ARC and RC models reduce to the SC model. Figure 2 shows the difference between real and estimated time to the most recent common ancestor (TMRCA) and the estimated mean mutation rate *μ* for both models, as well as the estimates of the parameter *ω* for the ARC model. The 95% credible intervals of both the TMRCA and *μ* almost always include the correct values of 0 and 5, respectively, but the intervals are slightly larger in the RC model for *μ* (mean length of 2.30 vs. 1.91), and much larger for the TMRCA (mean length of 24.82 vs. 10.91). This indicates that even if using the RC does not result in biased estimates, more precise estimates can be obtained using the ARC model, especially for dating nodes. The difference was less pronounced when simulation used lower values of *ω*, as expected since the ARC and RC models both reduce to the SC model when *ω *= 0, but even in these conditions the ARC presented a clear advantage in terms of precisely estimating the TMRCA (supplementary fig. S2, Supplementary Material online). The estimates of *ω* under the ARC model follow the true values of *ω* used in the simulation, which is as expected when the same model is used for simulation and inference but also shows that there is significant statistical power, even in these relatively small data sets, to correctly infer the level of relaxation of the molecular clock.

We applied treedater (Volz and Frost 2017) to the same data sets using the ARC model and computed parametric bootstrap values for the TMRCA, mean mutation rate *μ*, and relaxation parameter *ω* (supplementary fig. S3, Supplementary Material online). The inferred values of *ω* followed the correct values used in the simulations, which is as expected since the ARC model was used for both simulation and inference. The TMRCA and *μ* were correctly inferred with no evidence of bias, but the 95% confidence intervals estimated using parametric bootstrapping were wider than the Bayesian credible intervals in BactDating, which is certainly the result of inherent differences between these two statistical approaches rather than differences between the continuous and discrete models.

**Fig. 3.**
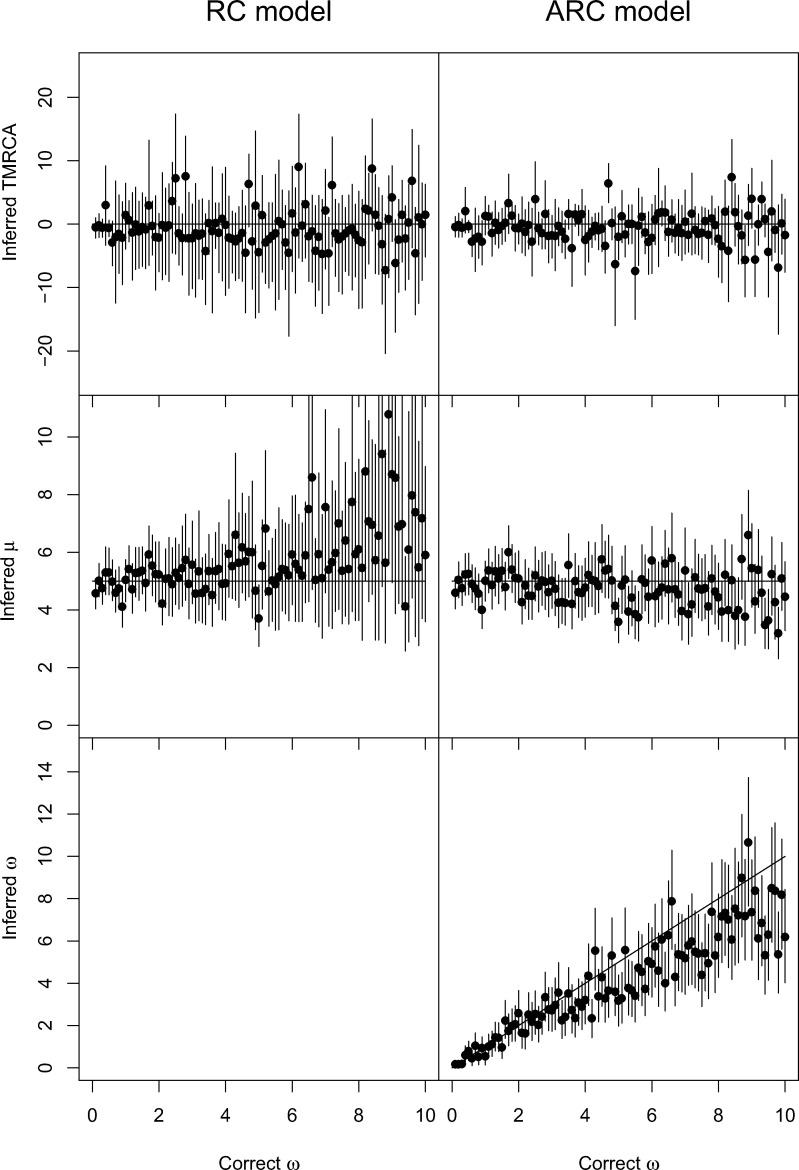
Application of BEAST2 to 100 simulated data sets. On the left, inference used the RC model and on the right, the new ARC model. The top row shows inferred values of the TMRCA (relative to the correct value), the middle row shows inferred values of the mean mutation rate *μ*, and the bottom row shows inferred values of the relaxation parameter *ω* for the ARC model. In each plot, the *x*-axis represents the value of *ω* used in the simulations (varied between 0 and 10) and the *y*-axis represents the inferred values, with a dot for the posterior mean and a bar for the 95% credible interval.

We applied BEAST2 (Bouckaert et al. 2019) to the same genome data sets using our new BEAST2 package. Inference was performed in BEAST2 using both the previous uncorrelated lognormal relaxed molecular clock (Drummond et al. 2006) and our new ARC model (fig. 3). We found that the inference of both the TMRCA and the mean clock rate *μ* was improved when using the ARC model. The estimates for both models were usually centered on the correct values, but the credible intervals for the RC model were much wider than for the ARC model for both the mean clock rate (mean lengths 4.12 vs. 2.01) and the TMRCA (mean lengths 14.29 vs. 8.08). There was a slight underestimation of the relaxation parameter *ω* for values >3, which reflects the difficulty to infer this parameter precisely and our choice of a conservative prior Gamma(0.1,1) with mean and variance equal to 0.1.

We also performed in BEAST2 another set of analyses in which we purposefully misspecified the mutation model by using an infinite site model for the simulations and a finite site model for the inference (supplementary fig. S4, Supplementary Material online). We used sequences of length only 1,000 bp to accentuate the difference between simulation and inference models, with all other conditions as before. This resulted in a relatively small bias when inferring with the ARC model, with a slight overestimation of both the mean rate *μ* and the relaxation parameter *ω*. However, the results with the uncorrelated lognormal relaxed molecular clock were worse in terms of both the clock rate and TMRCA estimates (supplementary fig. S4, Supplementary Material online). These results therefore show that the ARC model is fairly robust to model misspecification, with relatively little effect on the estimates of lineage dates.

### Application to Real Data Sets

We reanalyzed a previously published data set (Schuenemann et al. 2013) consisting of ten modern genomes plus five ancient genomes of *Mycobacterium leprae*, the causative agent of leprosy. In a previous analysis using BactDating (Didelot et al. 2018), the cSC model was found to be preferred to the cRC model using a reversible jump Markov Chain Monte-Carlo (rjMCMC; Green 1995) to compare between the two models which resulted in a Bayes factor of 141.85 in favor of cSC. We repeated this analysis using a similar rjMCMC to compare between cSC and our new cARC, and found once again that cSC was preferred, with an estimated Bayes factor of 57.82. Thus, in this model, as previously concluded (Didelot et al. 2018), there is no evidence for relaxation of the clock rate, whether a cRC or cARC model is used, and the inferred dated phylogeny is therefore unchanged (supplementary fig. S5, Supplementary Material online).

We also reanalyzed another previously published data set (Holt et al. 2013) consisting of 155 Vietnamese genomes from the VN clade of the bacterial pathogen *Shigella sonnei*. A previous analysis using BEAST estimated the TMRCA to be 1,982 [1,978–1,986] (Holt et al. 2013) and a separate analysis using the cRC model of BactDating found a very similar estimate of 1,983.45 [1,977.99; 1,986.88] (Didelot et al. 2018). We repeated this second analysis using the cARC model, and found a more precise estimate for the TMRCA of 1,983.04 [1,979.58; 1,985.91] (supplementary fig. S6, Supplementary Material online). In the previous cRC analysis, the mean evolutionary rate was estimated to be μ=4.22 [3.66–4.85] substitutions per genome per year, whereas with the new cARC, it was slightly lower at 3.93 [3.36; 4.51] substitutions per genome per year with a relaxation parameter *ω* of 1.72 [1.11; 2.44]. We computed the DIC (Spiegelhalter et al. 2002) under both cRC and cARC and found them to be, respectively, equal to 2,008.33 and 1,782.69. This represents conclusive evidence that this data set is better explained by the new cARC model rather than the previous cRC model, and therefore that additivity is an important property to analyze this data set.

**Fig. 4.**
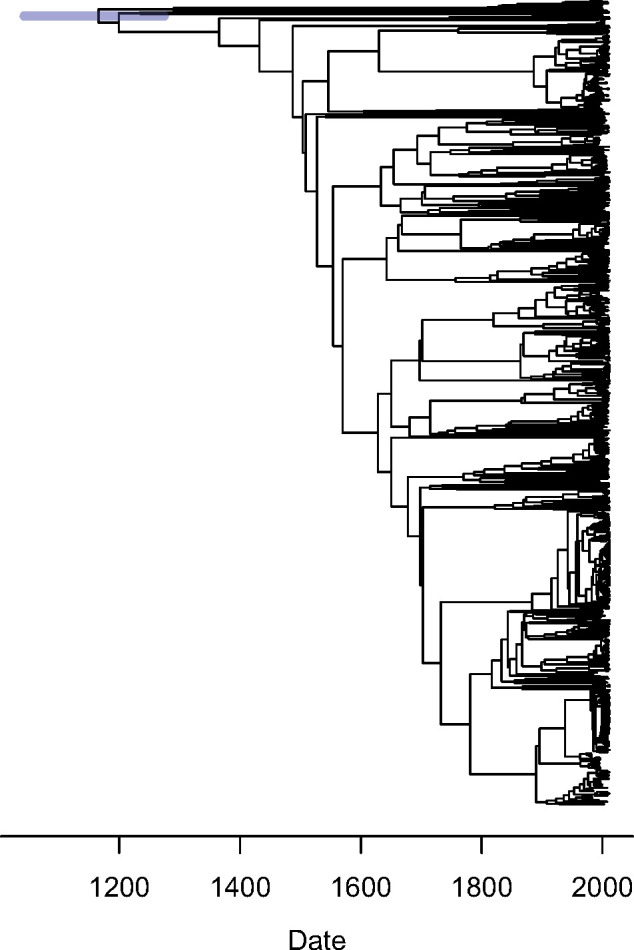
Application of the cARC model in BactDating to the Typhi data set. The inferred dated tree is shown with node positions on the *x*-axis representing the posterior mean date for each node and the blue bars representing the 95% credible intervals.

Finally, we present a new analysis of a question that has sparked debate for many years: the age of last common ancestor of Typhi, the serovar of *Salmonella enterica* which causes typhoid fever. This age was first estimated to be about 50 ka (Kidgell et al. 2002) based on a universal clock of $6\times 10^{-9}$ per site per year (Ochman and Wilson 1987; Ochman et al. 1999). This estimate was later revised to between 10 and 40 ka (Roumagnac et al. 2006), still based on the same universal clock. However, these estimates have been criticized on the basis that the universal clock is no longer believed to be valid (Morelli et al. 2010; Achtman 2016). Recent genomic studies on Typhi did not provide a new estimate for the age of Typhi, focusing instead on specific geographical regions or individual clades within Typhi (Wong et al. 2015, 2016; Britto et al. 2018; Park et al. 2018). One of these studies included a large number of genomes from the whole of Typhi, but reported a lack of temporal signal (Wong et al. 2015). This study therefore focused on the recently emerged H58 lineage of Typhi, within which they estimated a clock rate of 0.63 [0.59–0.67] substitutions per genome per year (Wong et al. 2015). We reanalyzed the 978 genomes from this study (Wong et al. 2015) which are not part of H58. We found, as reported by the authors, no evidence for a temporal signal on the basis of a linear regression of root-to-tip distances versus isolation dates (supplementary fig. S7, Supplementary Material online). However, this regression approach is not statistically powerful since root-to-tip distances are not independent from one another, and also because it makes an implicit assumption of a strict molecular clock. We therefore applied both the cRC and cARC models within BactDating, and found that cARC had a much smaller DIC of 11,107.77 compared with 17,419.52 for cRC. The cARC model is therefore supported by the data, and in this analysis, we estimate a mean rate *μ* of 0.38 [0.36; 0.42] substitutions per genome per year with relaxation parameter *ω* of 8.13 [7.16; 9.02]. This mean rate is similar to the previous estimate for H58 (Wong et al. 2015) which suggests that the temporal signal is correctly captured. On the other hand, this rate for the whole of Typhi is slightly lower than for the recent clade H58, which is consistent with the well-documented inverse relationship between estimated substitution rates and TMRCA (Ho and Larson 2006; Ho et al. 2011; Duchêne et al. 2014; Biek et al. 2015). We confirmed that this temporal signal under the cARC model is significant following a previously described method (Duchêne et al. 2015): The analysis was repeated 100 times with sampling dates randomized, and we found that the 95% credible interval of *μ* mentioned above did not overlap with any of the intervals obtained after randomization. Based on this analysis, with BactDating and the cARC model (fig. 4), our estimate of the age of Typhi is 1,166 CE [1,042.57; 1,274.37], which suggests that early estimates based on a universal clock were inaccurate, as previously mentioned (Morelli et al. 2010; Achtman 2016).

## Discussion

We defined the additivity property to be that the sum of the numbers of substitutions on n≥2 branches should have the same distribution as the number of substitutions on a single branch of length equal to the sum of the *n* branches. We showed that the existing SC models for both discrete (SC, eq. 1) and continuous (cSC, eq. 12) cases satisfy this additivity property, whereas commonly used uncorrelated RC models (RC, eq. 3 and cRC, eq. 14) do not. However, we have defined two new RC models for the discrete (ARC, eq. 10) and continuous cases (cARC, eq. 15) that satisfy the additivity property. We implemented the new relaxed additive models in three popular software for the inference of dated phylogenies, namely BactDating (Didelot et al. 2018), treedater (Volz and Frost 2017), and BEAST2 (Bouckaert et al. 2019). We have shown using simulated data sets that inference using nonadditive models could be misleading if the true underlying model is additive. We have also shown in real data sets that the additive models can provide better results than the previous nonadditive models, and can represent a better fit to the data. All the clock models we described belong to the class of uncorrelated RC models, where the rate of each branch is uncorrelated with the rate of nearby branches. An alternative class of models is the autocorrelated RC models, where neighboring branches share similar rates (Thorne et al. 1998; Ho et al. 2005; Ho and Duchêne 2014; Bromham et al. 2018). We focused on uncorrelated RC models rather than autocorrelated RC models because the former are much more frequently used in the field of genomic epidemiology.

It is interesting to note that all the additive models we described, whether strict or relaxed, and whether discrete or continuous, belong to the same class of stochastic processes. The SC model is a simple Poisson process on the branches of the phylogeny, whereas the ARC model corresponds to a negative binomial process (Barndorff-Nielsen and Yeo 1969; Kozubowski and Podgorski 2009). The cSC and cARC models both correspond to a gamma process, and these three processes are all Lévy processes, which means that they have stationary and independent increments (Applebaum 2004). Lévy processes generate infinitely divisible random variables, which implies the additivity property that we sought, since a branch may be divided into any number of parts when samples are added into a phylogenetic tree, and this division should not affect the distribution of the number of mutations on that branch. The ARC model in equation (10) can therefore be obtained by considering that branches are made of *L* infinitesimal units, each of which has an associated number of substitutions distributed as $\text{NegBin}\left(\frac{\mu l_{i}}{Ls},\frac{\omega }{1+\omega }\right)$. The sum of these *L* random variables corresponds to the number of substitutions on the whole branch, which is distributed as in equation (10) using the negative binomial summation rule (eq. 11). Likewise, the cARC model in equation (15) can be derived using $\text{Gamma}\left(\frac{\mu l_{i}}{L(1+\omega )},1+\omega \right)$ for the distribution of substitution of each infinitesimal unit and using the gamma summation rule (eq. 16).

One of the earliest proposed models for a relaxed molecular clock (Takahata 1987) was based on a compound Poisson process which is another type of Lévy process and therefore satisfied the additivity property, but this model has not been used in practice in a phylogenetic framework. More generally, Lévy processes are natural to describe biological phenomena in time, and have been proposed several times recently to model evolutionary jumps (Jourdain et al. 2012; Landis et al. 2013; Duchen et al. 2017), which is similar to the relaxation of the molecular clock we want to model in this study. In conclusion, we recommend using additive RC models when performing genomic epidemiology studies based on the estimation of dated phylogenies, as these models are sounder than previously used models, both statistically and biologically.

## Materials and Methods

### Simulated Data Sets

The simulated data sets were generated by first sampling from the heterochronous coalescent model (Drummond et al. 2002) with an expected coalescent time for any two lineages equal to $\alpha =N_{e}g=5$ years, where *N*_e_ is the effective population size and *g* is the duration of a generation. For each branch duration *l_i_*, we simulated a mutation rate *m_i_* using equation (9) for a given value of the mutation rate *μ* and relaxation parameter *ω* of the ARC model. The software tool seq-gen (Rambaut and Grass 1997) was then applied to generate genomes of length 10,000 bp assuming a Jukes–Cantor model. For the application of BactDating and treedater to the simulated data sets, we first reconstructed a phylogeny using PhyML (Guindon et al. 2010). For the application of BEAST2, the sequence data were used directly as input. For the simulation of data under an infinite site model (supplementary fig. S4, Supplementary Material online), we sampled the number of mutations *x_i_* for each branch of length *l_i_* from equation (10) and applied these mutations to sequences of length 1,000 bp. All the data and code used to generate and analyze these simulations are available at https://github.com/xavierdidelot/ARC-examples.

### Implementation and Availability

In order to make our new additive RC models as readily available as possible, we have implemented them in three separate preexisting software packages for the inference of dated phylogenies.

The treedater software can perform dating of the nodes of a phylogeny using maximum likelihood (Volz and Frost 2017). The SC and RC models were previously implemented in treedater, and we have extended it with the new ARC model (eq. 10). treedater is an R package available at https://github.com/emvolz/treedater.

The BactDating software can perform dating of the nodes of a phylogeny using Bayesian inference (Didelot et al. 2018). The SC, RC, cSC, and cRC models were previously implemented in BactDating, and we have extended it with the new ARC and cARC models (eqs. 10 and 15). Furthermore, BactDating can simulate data sets based on all six clock models described above. BactDating is an R package available at https://github.com/xavierdidelot/BactDating.

The BEAST2 (Bouckaert et al. 2019) software can infer dated phylogenies directly from the genetic data. Both a SC model and an uncorrelated RC model (Drummond et al. 2006) were previously implemented in BEAST2, and we have created a BEAST2 package so that it can now use the new ARC model. This BEAST2 package is available at https://github.com/igococha/ARC. This new implementation is based directly on the model in equation (9) for the per-branch evolutionary rates, as opposed to the models implemented in treedater and BactDating which are based on branch lengths. This difference in implementation is due to the fact that BEAST does not explicitly model the numbers *x_i_* of mutations on each branch, but instead considers the products of the branch rates and durations to compute the probability of a data set using the pruning algorithm (Felsenstein 1981).

When analyzing data under the new ARC model, it is necessary to infer the new relaxation parameter *ω* jointly with the other parameters such as the mean clock rate *μ* and the dates of the nodes. For maximum likelihood inference in treedater, we simply optimize this new parameter along with the others in the same way as for example the mean clock rate *μ*. For Bayesian inference in BactDating and BEAST2, we use an additional Metropolis–Hastings move for *ω* assuming a gamma prior with user-specified parameters.

## Supplementary Material

Supplementary data are available at *Molecular Biology and Evolution* online.

## Acknowledgments

We acknowledge funding from the Medical Research Council (grants MR/N010760/1 and MR/R015600/1) and the National Institute for Health Research (NIHR) Health Protection Research Unit in Genomics and Enabling Data.

## Supplementary Material

msaa193_Supplementary_Data

## References

[msaa193-B1] Achtman M. 2016. How old are bacterial pathogens? Proc R Soc B. 283(1836):20160990. 10.1098/rspb.2016.0990 PMC5013766 27534956

[msaa193-B2] Applebaum D. 2004. Lévy processes – from probability to finance and quantum groups. Not AMS. 51:1336–1347.

[msaa193-B3] Barndorff-Nielsen O, Yeo GF. 1969. Negative binomial processes. J Appl Probab. 6(3):633–647.

[msaa193-B4] Biek R, Pybus OG, Lloyd-Smith JO, Didelot X. 2015. Measurably evolving pathogens in the genomic era. Trends Ecol Evol. 30(6):306–313. 25887947 10.1016/j.tree.2015.03.009 PMC4457702

[msaa193-B5] Bouckaert R, Vaughan TG, Barido-Sottani J, Duchêne S, Fourment M, Gavryushkina A, Heled J, Jones G, Kühnert D, De Maio N, et al 2019. BEAST 2.5: an advanced software platform for Bayesian evolutionary analysis. PLoS Comput Biol. 15(4):e1006650. 30958812 10.1371/journal.pcbi.1006650 PMC6472827

[msaa193-B6] Britto CD, Dyson ZA, Duchene S, Carter MJ, Gurung M, Kelly DF, Murdoch DR, Ansari I, Thorson S, Shrestha S, et al 2018. Laboratory and molecular surveillance of paediatric typhoidal *Salmonella* in Nepal: antimicrobial resistance and implications for vaccine policy. PLoS Negl Trop Dis. 12:1–19. 10.1371/journal.pntd.0006408 PMC5933809 29684021

[msaa193-B7] Bromham L, Duchene S, Hua X, Ritchie A, Duchene D, Ho S. 2018. Bayesian molecular dating: opening up the black box. Biol Rev. 93(2):1165–1191. 29243391 10.1111/brv.12390

[msaa193-B8] Brooks SPB, Gelman AG. 1998. General methods for monitoring convergence of iterative simulations. J Comput Graph Stat. 7(4):434–455.

[msaa193-B9] Carroll MW, Matthews DA, Hiscox JA, Elmore MJ, Pollakis G, Rambaut A, Hewson R, García-Dorival I, Bore JA, Koundouno R, et al 2015. Temporal and spatial analysis of the 2014-2015 Ebola virus outbreak in West Africa. Nature 524(7563):97–101. 26083749 10.1038/nature14594 PMC10601607

[msaa193-B10] Didelot X, Croucher NJ, Bentley SD, Harris SR, Wilson DJ. 2018. Bayesian inference of ancestral dates on bacterial phylogenetic trees. Nucleic Acids Res. 46(22):e134. 30184106 10.1093/nar/gky783 PMC6294524

[msaa193-B11] Didelot X, Fraser C, Gardy J, Colijn C. 2017. Genomic infectious disease epidemiology in partially sampled and ongoing outbreaks. Mol Biol Evol. 34(4):997–1007. 28100788 10.1093/molbev/msw275 PMC5850352

[msaa193-B12] Dinh V, Darling AE, Matsen FA. 2018. Online Bayesian phylogenetic inference: theoretical foundations via sequential Monte Carlo. Syst Biol. 67(3):503–517. 29244177 10.1093/sysbio/syx087 PMC5920340

[msaa193-B13] Drummond AJ, Ho SYW, Phillips MJ, Rambaut A. 2006. Relaxed phylogenetics and dating with confidence. PLoS Biol. 4(5):e88. 16683862 10.1371/journal.pbio.0040088 PMC1395354

[msaa193-B14] Drummond AJ, Nicholls GK, Rodrigo AG, Solomon W. 2002. Estimating mutation parameters, population history and genealogy simultaneously from temporally spaced sequence data. Genetics 161(3):1307–1320. 12136032 10.1093/genetics/161.3.1307 PMC1462188

[msaa193-B15] Drummond AJ, Suchard MA. 2010. Bayesian random local clocks, or one rate to rule them all. BMC Biol. 8:114. 20807414 10.1186/1741-7007-8-114 PMC2949620

[msaa193-B16] Duchen P, Leuenberger C, Szilágyi SM, Harmon L, Eastman J, Schweizer M, Wegmann D. 2017. Inference of evolutionary jumps in large phylogenies using Levy processes. Syst Biol. 66(6):950–963. 28204787 10.1093/sysbio/syx028 PMC5790141

[msaa193-B17] Duchêne S, Duchêne D, Holmes EC, Ho SY. 2015. The performance of the date-randomization test in phylogenetic analyses of time-structured virus data. Mol Biol Evol. 32(7):1895–1906. 25771196 10.1093/molbev/msv056

[msaa193-B18] Duchêne S, Holmes EC, Ho SYW. 2014. Analyses of evolutionary dynamics in viruses are hindered by a time-dependent bias in rate estimates. Proc R Soc B. 281(1786):20140732. 10.1098/rspb.2014.0732 PMC4046420 24850916

[msaa193-B19] Faria NR, Quick J, Claro IM, Thézé J, De Jesus JG, Giovanetti M, Kraemer MU, Hill SC, Black A, Da Costa AC, et al 2017. Establishment and cryptic transmission of Zika virus in Brazil and the Americas. Nature 546(7658):406–410. 28538727 10.1038/nature22401 PMC5722632

[msaa193-B20] Felsenstein J. 1981. Evolutionary trees from DNA sequences: a maximum likelihood approach. J Mol Evol. 17(6):368–376. 7288891 10.1007/BF01734359

[msaa193-B21] Fourment M, Claywell BC, Dinh V, McCoy C, Matsen FA, Darling AE. 2018. Effective online Bayesian phylogenetics via sequential Monte Carlo with guided proposals. Syst Biol. 67(3):490–502. 29186587 10.1093/sysbio/syx090 PMC5920299

[msaa193-B22] Gelman A, Rubin DB. 1992. Inference from iterative simulation using multiple sequences. Stat Sci. 7(4):457–511.

[msaa193-B23] Gill MS, Lemey P, Suchard MA, Rambaut A, Baele G. 2020. Online Bayesian phylodynamic inference in BEAST with application to epidemic reconstruction. Mol Biol Evol. 37(6):1832–1842. 32101295 10.1093/molbev/msaa047 PMC7253210

[msaa193-B24] Green PJ. 1995. Reversible jump Markov chain Monte Carlo computation and Bayesian model determination. Biometrika 82(4):711–732.

[msaa193-B25] Guindon S, Dufayard JF, Lefort V, Anisimova M, Hordijk W, Gascuel O. 2010. New algorithms and methods to estimate maximum-likelihood phylogenies: assessing the performance of PhyML 3.0. Syst Biol. 59(3):307–321. 20525638 10.1093/sysbio/syq010

[msaa193-B26] Hadfield J, Megill C, Bell SM, Huddleston J, Potter B, Callender C, Sagulenko P, Bedford T, Neher RA. 2018. NextStrain: real-time tracking of pathogen evolution. Bioinformatics 34(23):4121–4123. 29790939 10.1093/bioinformatics/bty407 PMC6247931

[msaa193-B27] Ho SY, Phillips MJ, Drummond AJ, Cooper A. 2005. Accuracy of rate estimation using relaxed-clock models with a critical focus on the early metazoan radiation. Mol Biol Evol. 22(5):1355–1363. 15758207 10.1093/molbev/msi125

[msaa193-B28] Ho SYW, Duchêne S. 2014. Molecular-clock methods for estimating evolutionary rates and timescales. Mol Ecol. 23(24):5947–5965. 25290107 10.1111/mec.12953

[msaa193-B29] Ho SYW, Lanfear R, Bromham L, Phillips MJ, Soubrier J, Rodrigo AG, Cooper A. 2011. Time-dependent rates of molecular evolution. Mol Ecol. 20(15):3087–3101. 21740474 10.1111/j.1365-294X.2011.05178.x

[msaa193-B30] Ho SYW, Larson G. 2006. Molecular clocks: when times are a-changin’. Trends Genet. 22(2):79–83. 16356585 10.1016/j.tig.2005.11.006

[msaa193-B31] Ho SYW, Shapiro B. 2011. Skyline-plot methods for estimating demographic history from nucleotide sequences. Mol Ecol Resour. 11(3):423–434. 21481200 10.1111/j.1755-0998.2011.02988.x

[msaa193-B32] Holt KE, Thieu Nga TV, Thanh DP, Vinh H, Kim DW, Vu Tra MP, Campbell JI, Hoang NVM, Vinh NT, Minh PV, et al 2013. Tracking the establishment of local endemic populations of an emergent enteric pathogen. Proc Natl Acad Sci U S A. 110(43):17522–17527. 24082120 10.1073/pnas.1308632110 PMC3808646

[msaa193-B33] Jones BR, Poon AF. 2017. Node.dating: dating ancestors in phylogenetic trees in R. Bioinformatics 33(6):932–934. 28365756 10.1093/bioinformatics/btw744 PMC5860581

[msaa193-B34] Jourdain B, Méléard S, Woyczynski WA. 2012. Lévy flights in evolutionary ecology. J Math Biol. 65(4):677–707. 22002665 10.1007/s00285-011-0478-5

[msaa193-B35] Kidgell C, Reichard U, Wain J, Linz B, Torpdahl M, Dougan G, Achtman M. 2002. *Salmonella typhi*, the causative agent of typhoid fever, is approximately 50,000 years old. Infect Genet Evol. 2(1):39–45. 12797999 10.1016/s1567-1348(02)00089-8

[msaa193-B36] Kozubowski T, Podgorski K. 2009. Distributional properties of the negative binomial Lévy process. Probab Math Stat. 29:43–71.

[msaa193-B37] Kumar S. 2005. Molecular clocks: four decades of evolution. Nat Rev Genet. 6(8):654–662. 16136655 10.1038/nrg1659

[msaa193-B38] Landis MJ, Schraiber JG, Liang M. 2013. Phylogenetic analysis using Lévy processes: finding jumps in the evolution of continuous traits. Syst Biol. 62(2):193–204. 23034385 10.1093/sysbio/sys086 PMC3566600

[msaa193-B39] Lartillot N, Phillips MJ, Ronquist F. 2016. A mixed relaxed clock model. Philos Trans R Soc B. 371(1699):20150132. 10.1098/rstb.2015.0132 PMC4920333 27325829

[msaa193-B40] Lepage T, Bryant D, Philippe H, Lartillot N. 2007. A general comparison of relaxed molecular clock models. Mol Biol Evol. 24(12):2669–2680. 17890241 10.1093/molbev/msm193

[msaa193-B41] Liò P, Goldman N. 1998. Models of molecular evolution and phylogeny. Genome Res. 8(12):1233–1244. 9872979 10.1101/gr.8.12.1233

[msaa193-B42] Morelli G, Didelot X, Kusecek B, Schwarz S, Bahlawane C, Falush D, Suerbaum S, Achtman M. 2010. Microevolution of *Helicobacter pylori* during prolonged infection of single hosts and within families. PLoS Genet. 6(7):e1001036. 20661309 10.1371/journal.pgen.1001036 PMC2908706

[msaa193-B43] Nguyen LT, Schmidt HA, Von Haeseler A, Minh BQ. 2015. IQ-TREE: a fast and effective stochastic algorithm for estimating maximum-likelihood phylogenies. Mol Biol Evol. 32(1):268–274. 25371430 10.1093/molbev/msu300 PMC4271533

[msaa193-B44] Ochman H, Elwyn S, Moran N. 1999. Calibrating bacterial evolution. Proc Natl Acad Sci U S A. 96(22):12638–12643. 10535975 10.1073/pnas.96.22.12638 PMC23026

[msaa193-B45] Ochman H, Wilson AC. 1987. Evolution in bacteria: evidence for a universal substitution rate in cellular genomes. J Mol Evol. 26(1-2):74–86. 3125340 10.1007/BF02111283

[msaa193-B46] Park SE, Pham DT, Boinett C, Wong VK, Pak GD, Panzner U, Espinoza LMC, von Kalckreuth V, Im J, Schütt-Gerowitt H, et al 2018. The phylogeography and incidence of multi-drug resistant typhoid fever in sub-Saharan Africa. Nat Commun. 9:5094. 30504848 10.1038/s41467-018-07370-z PMC6269545

[msaa193-B47] Plummer M, Best N, Cowles K, Vines K. 2006. CODA: convergence diagnosis and output analysis for. MCMC R News. 6:7–11.

[msaa193-B48] Price MN, Dehal PS, Arkin AP. 2010. FastTree 2–approximately maximum-likelihood trees for large alignments. PLoS One 5(3):e9490. 20224823 10.1371/journal.pone.0009490 PMC2835736

[msaa193-B49] Quick J, Loman NJ, Duraffour S, Simpson JT, Severi E, Cowley L, Bore JA, Koundouno R, Dudas G, Mikhail A, et al 2016. Real-time, portable genome sequencing for Ebola surveillance. Nature 530(7589):228–232.. 26840485 10.1038/nature16996 PMC4817224

[msaa193-B50] Rambaut A, Grass NC. 1997. Seq-Gen: an application for the Monte Carlo simulation of DNA sequence evolution along phylogenetic trees. Bioinformatics 13(3):235–238. 10.1093/bioinformatics/13.3.235 9183526

[msaa193-B51] Roumagnac P, Weill F-X, Dolecek C, Baker S, Brisse S, Chinh NT, Le TAH, Acosta CJ, Farrar J, Dougan G, et al 2006. Evolutionary history of *Salmonella typhi*. Science 314(5803):1301–1304. 17124322 10.1126/science.1134933 PMC2652035

[msaa193-B52] Sagulenko P, Puller V, Neher RA. 2018. TreeTime: maximum likelihood phylodynamic analysis. Virus Evol. 4(1):vex042. 29340210 10.1093/ve/vex042 PMC5758920

[msaa193-B53] Schuenemann VJ, Singh P, Mendum TA, Krause-Kyora B, Jäger G, Bos KI, Herbig A, Economou C, Benjak A, Busso P, et al 2013. Genome-wide comparison of medieval and modern *Mycobacterium leprae*. Science 341(6142):179–183. 23765279 10.1126/science.1238286

[msaa193-B54] Spiegelhalter D, Best N, Carlin B, Van der Linde A. 2002. Bayesian measures of model complexity and fit. J R Stat Soc B. 64(4):583–639.

[msaa193-B55] Stamatakis A. 2014. RAxML version 8: a tool for phylogenetic analysis and post-analysis of large phylogenies. Bioinformatics 30(9):1312–1313. 24451623 10.1093/bioinformatics/btu033 PMC3998144

[msaa193-B56] Suchard MA, Lemey P, Baele G, Ayres DL, Drummond AJ, Rambaut A. 2018. Bayesian phylogenetic and phylodynamic data integration using BEAST 1.10. Virus Evol. 4(1):vey016. 29942656 10.1093/ve/vey016 PMC6007674

[msaa193-B57] Takahata N. 1987. On the overdispersed molecular clock. Genetics 116(1):169–179. 3596230 10.1093/genetics/116.1.169 PMC1203115

[msaa193-B58] Thorne JL, Kishino H, Painter IS. 1998. Estimating the rate of evolution of the rate of molecular evolution. Mol Biol Evol. 15(12):1647–1657. 9866200 10.1093/oxfordjournals.molbev.a025892

[msaa193-B59] To TH, Jung M, Lycett S, Gascuel O. 2016. Fast dating using least-squares criteria and algorithms. Syst Biol. 65(1):82–97. 26424727 10.1093/sysbio/syv068 PMC4678253

[msaa193-B60] Volz EM, Frost SDW. 2017. Scalable relaxed clock phylogenetic dating. Virus Evol. 3:vex025.

[msaa193-B61] Volz EM, Koelle K, Bedford T. 2013. Viral phylodynamics. PLoS Comput Biol. 9(3):e1002947. 23555203 10.1371/journal.pcbi.1002947 PMC3605911

[msaa193-B62] Volz EM, Wiuf C, Grad YH, Frost SDW, Dennis AM, Didelot X. 2020. Identification of hidden population structure in time-scaled phylogenies. Syst Biol. doi: 10.1093/sybio/syaa009. 10.1093/sysbio/syaa009 PMC8559910 32049340

[msaa193-B63] Wong VK, Baker S, Connor TR, Pickard D, Page AJ, Dave J, Murphy N, Holliman R, Sefton A, Millar M, et al 2016. An extended genotyping framework for *Salmonella enterica* serovar Typhi, the cause of human typhoid. Nat Commun. 7:1–11. 10.1038/ncomms12827 PMC5059462 27703135

[msaa193-B64] Wong VK, Baker S, Pickard DJ, Parkhill J, Page AJ, Feasey NA, Kingsley RA, Thomson NR, Keane JA, Weill FX, et al 2015. Phylogeographical analysis of the dominant multidrug-resistant H58 clade of *Salmonella* Typhi identifies inter- and intracontinental transmission events. Nat Genet. 47(6):632–639. 25961941 10.1038/ng.3281 PMC4921243

[msaa193-B65] Yang Z, Rannala B. 2012. Molecular phylogenetics: principles and practice. Nat Rev Genet. 13(5):303–314. 22456349 10.1038/nrg3186

[msaa193-B66] Zuckerkandl E, Pauling L. 1962. Molecular disease, evolution, and genic heterogeneity. In: Kasha M, Pullman B, editors. Horizons Biochem. New York: Academic Press. p. 189–222.

