# Supplementary material for: Additive Uncorrelated Relaxed Clock Models for the Dating of Genomic Epidemiology Phylogenies
Source: Mol Biol Evol. 2020 Jul 28;38(1):307–17. doi: 10.1093/molbev/msaa193 (PMC8480190; doi:10.1093/molbev/msaa193)
Supplement: msaa193_Supplementary_Data [file msaa193_Supplementary_Data.pdf]

Supplementary Material

Additive uncorrelated relaxed clock models for the dating of genomic epidemiology phylogenies

Xavier Didelot[1,*], Igor Siveroni[2] and Erik M Volz[2]

[1] School of Life Sciences and Department of Statistics, University of Warwick, United Kingdom

[2] Department of Infectious Disease Epidemiology, School of Public Health, Imperial College London, United Kingdom

[*] Corresponding author. Tel: 0044 (0)2476 572827. Email: xavier.didelot@gmail.com

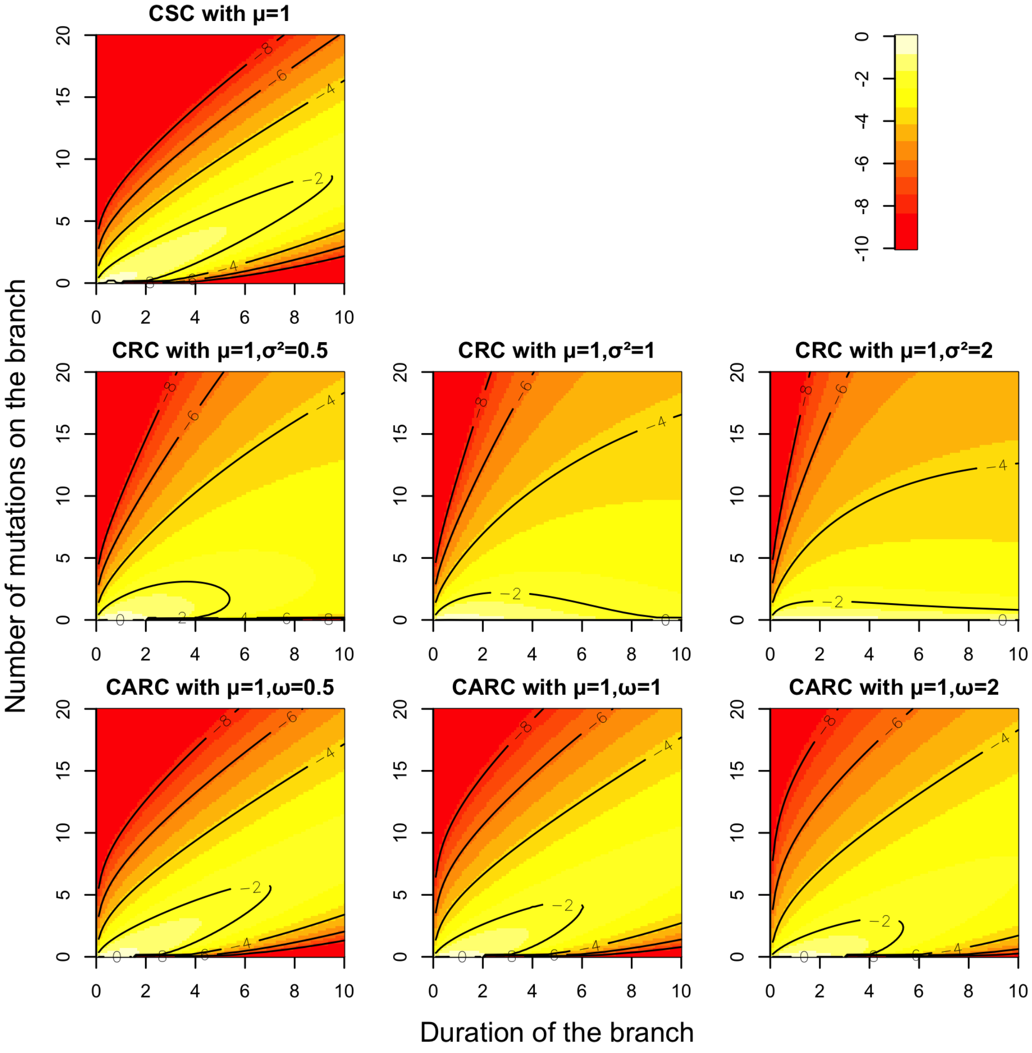

Figure S1: **Comparison of clock models for continuous branch lengths.** The top-left plot shows the cSC model, with $\mu = 1$. The second row shows the cRC model, with $\mu = 1$ and $\sigma^2 = 0.5$, 1 and 2 respectively from left to right. The third row shows the cARC model, with $\mu = 1$ and $\omega = 0.5$, 1 and 2 respectively from left to right. In each plot, the x-axis shows values of l_i, the y-axis shows values of x_i and the color represents the value of the log of $p(x_i|l_i)$ as per the legend.

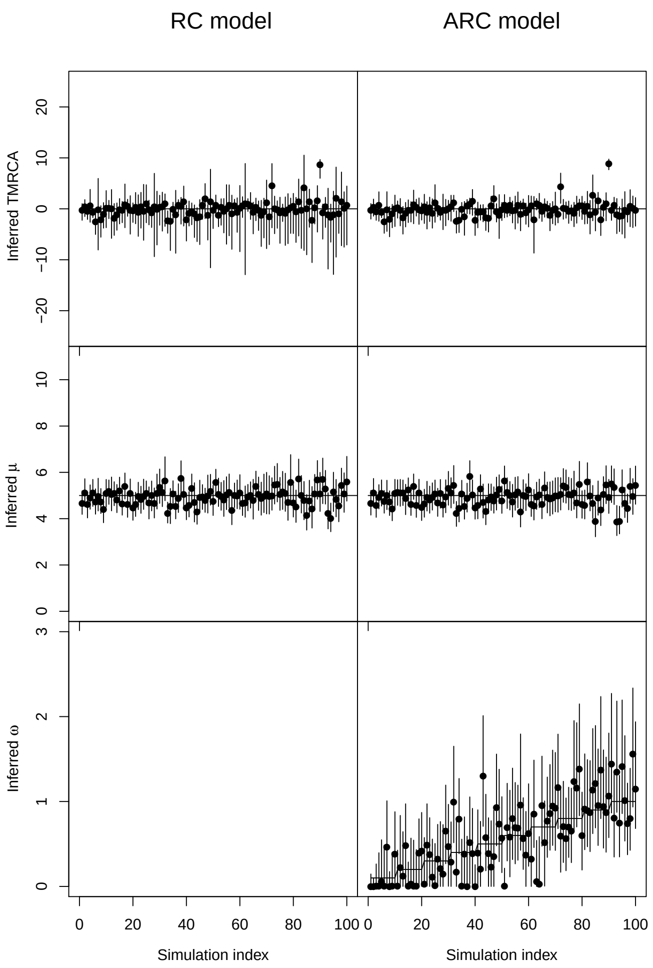

Figure S2: **Application of BactDating to 100 simulated datasets.** On the left inference used the RC model and on the right the ARC model. The top row shows inferred values of the TMRCA (relative to the correct value), the middle row shows inferred values of the mean mutation rate μ, and the bottom row shows inferred values of the relaxation parameter ω for the ARC model. In each plot, the x-axis represents the index of the simulations and the y-axis represents the inferred values, with a dot for the posterior mean and a bar for the 95% credible interval. The correct value of ω was 0.1 in the simulations with index 1 to 10, 0.2 in the simulations with index 11 to 20, etc until simulations with index 91 to 100 which had a value of $\omega = 1$.

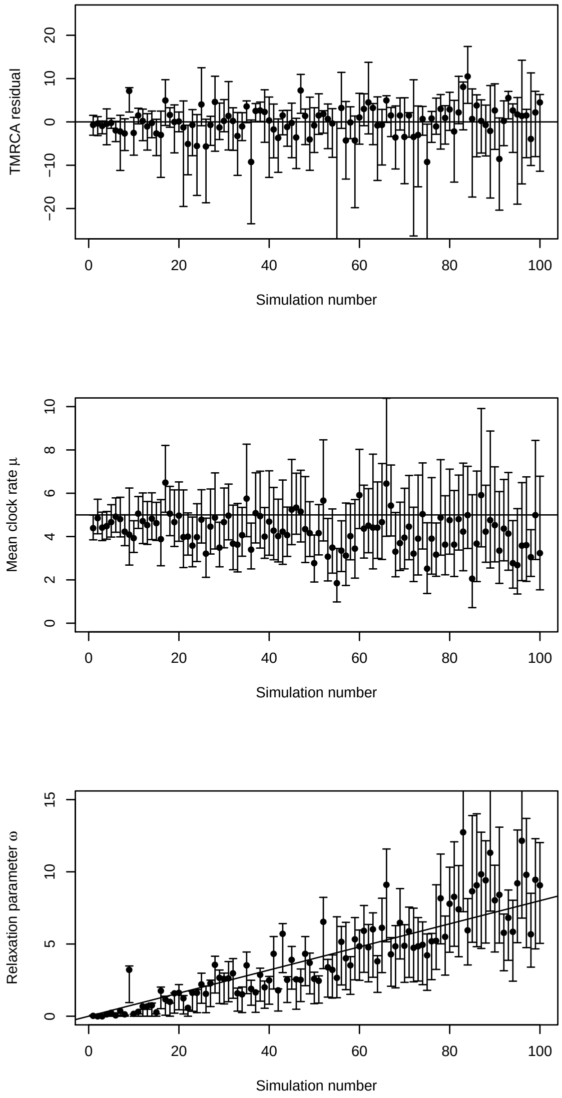

Figure S3: **Application of the ARC model in treedater to 100 simulated datasets.** The top, middle and bottom rows show inferred values of the TMRCA, mean mutation rate μ and relaxation parameter ω, respectively.

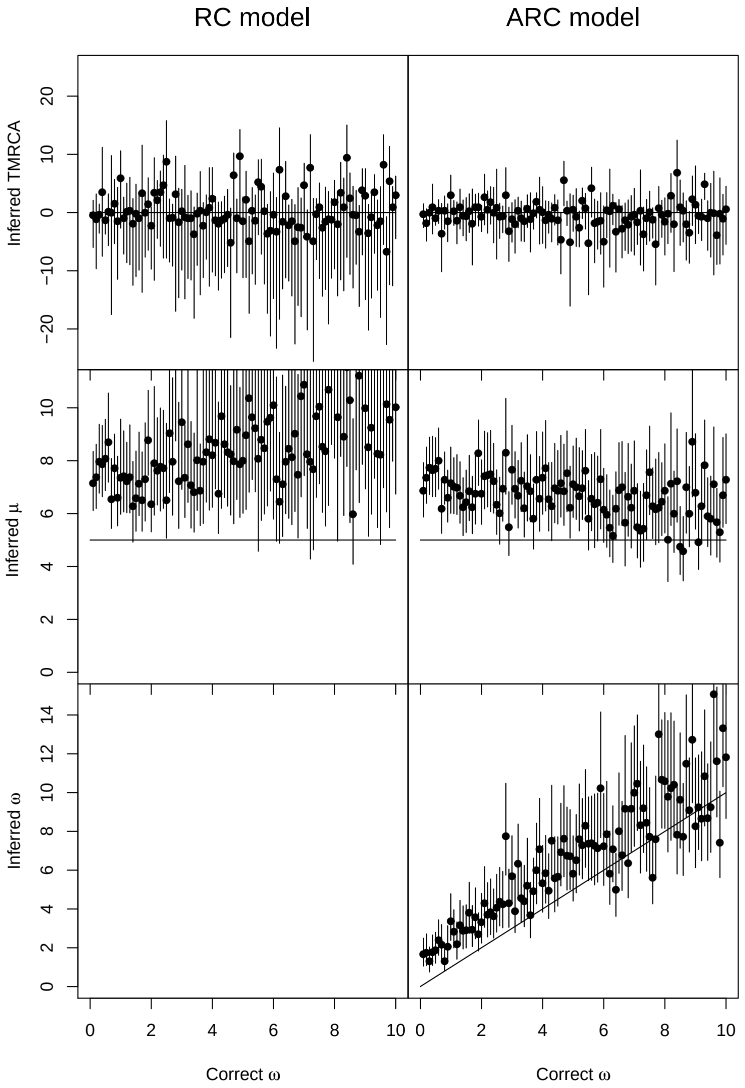

Figure S4: **Application of BEAST2 to 100 datasets simulated under an infinite site model.** On the left inference used the RC model and on the right the new ARC model. The top row shows inferred values of the TMRCA (relative to the correct value), the middle row shows inferred values of the mean mutation rate μ, and the bottom row shows inferred values of the relaxation parameter ω for the ARC model. In each plot, the x-axis represents the value of ω used in the simulations (varied between 0 and 10) and the y-axis represents the inferred values, with a dot for the posterior mean and a bar for the 95% credible interval.

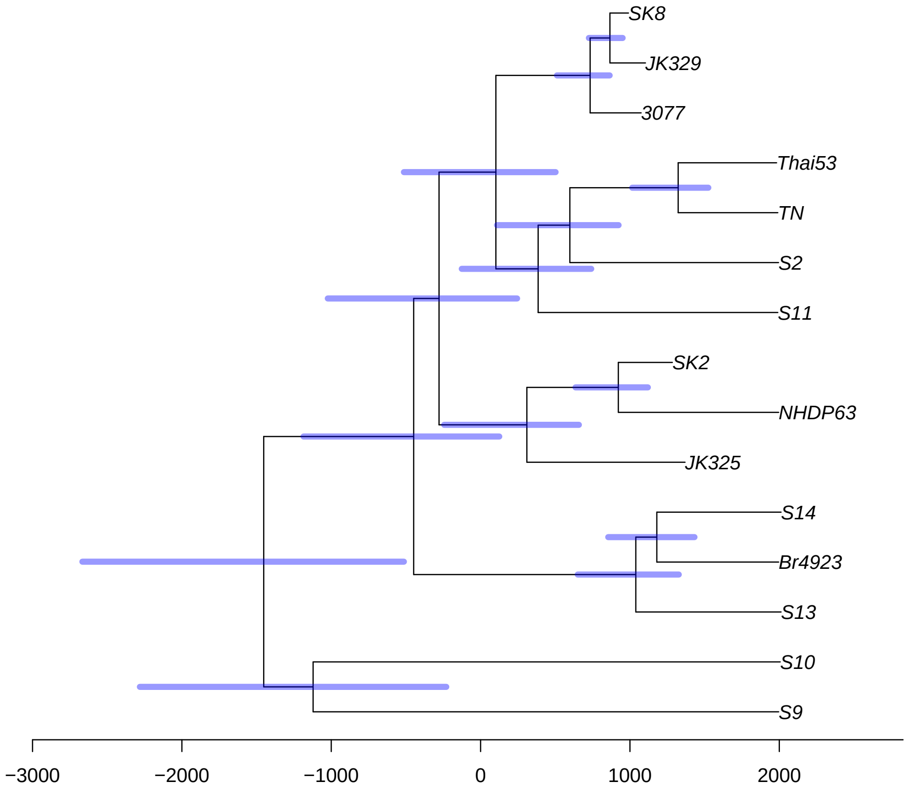

Figure S5: **Dated phylogeny for the dataset of *M. leprae*.**

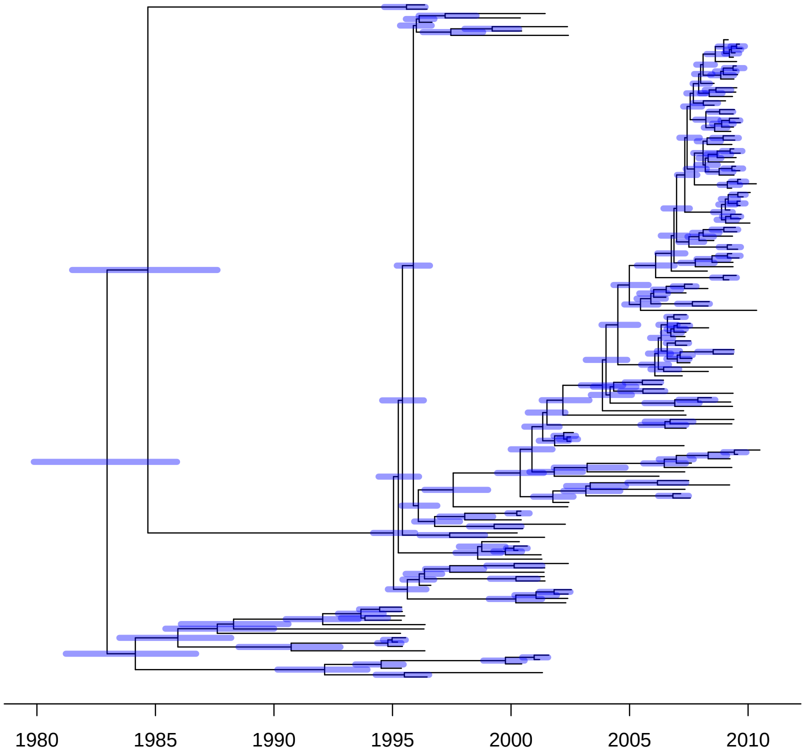

Figure S6: **Dated phylogeny for the dataset of *S. sonnei*.**

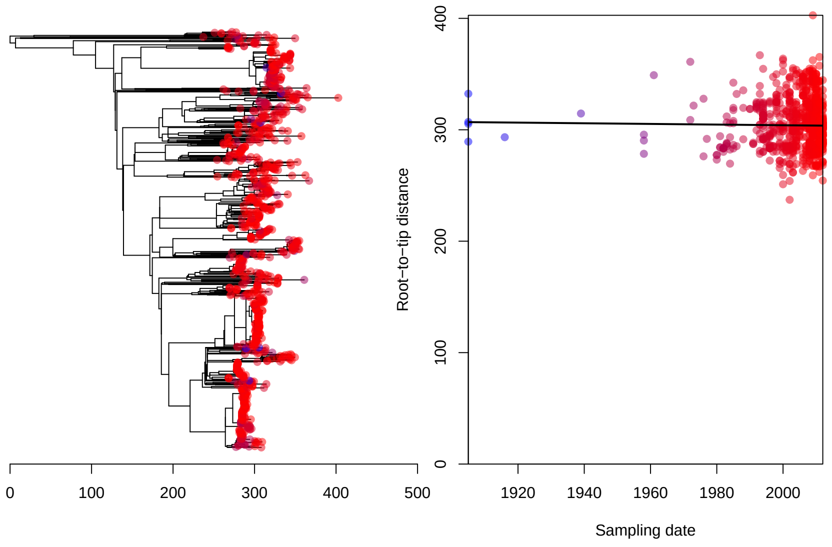

Figure S7: **Root-to-tip regression for Typhi.** Linear regression of the root-to-tip distances against isolation dates for the Typhi dataset.
